# 2238. Effect of Cefazolin Breakpoint Update in Susceptibility Reports on Antimicrobial Use

**DOI:** 10.1093/ofid/ofad500.1860

**Published:** 2023-11-27

**Authors:** Valerie Buckley, R Scott Ferren, Noor Zaidan, Rachel S Britt, David Reynoso

**Affiliations:** UTMB, Houston, Texas; UTMB, Houston, Texas; UTMB, Houston, Texas; UTMB, Houston, Texas; The University of Texas Medical Branch, Galveston, Texas

## Abstract

**Background:**

The Clinical Laboratory Standards Institute updated the breakpoints for cefazolin in the treatment of *E. coli, Klebsiella*, and *Proteus* (EKP) infections in 2011 to reflect doses used in clinical practice and achievable therapeutic concentrations in different body sites (urine vs. non-urine). The UTMB clinical microbiology laboratory adopted this update in January 2022 with the introduction of MicroScan™ technology. Our objective was to evaluate the effect of the cefazolin breakpoint update on antimicrobial use in EKP bloodstream infections.

**Methods:**

This was a retrospective, multicenter, pre-post, study conducted at four UTMB hospitals. Pre-intervention data was collected from January 2021 to December 2021 and post-intervention data was collected from February 2022 to March 2023. Patients ≥ 18 years old were included if they were admitted to a UTMB hospital and received antibiotics for EKP bacteremia. Texas Department of Criminal Justice patients, those with beta-lactam anaphylaxis, polymicrobial bacteremia, immunocompromised patients, and *Klebsiella aerogenes* isolates were excluded. The primary outcome was cefazolin days of therapy (DOTs) for EKP bacteremia. Extended-spectrum beta-lactamase (ESBL)-producing isolates and *Clostridioides difficile* (*C. difficile*) infection six months after the index admission, and in-hospital and 28-day mortality were also assessed.

**Results:**

There were 50 and 48 patients included in the pre- and post-intervention periods, respectively. Most patients were female (59%) with a mean age of 67.8 years (standard deviation [SD] 14.9). The most isolated gram-negative pathogen was *Klebsiella pneumonia* (81%) followed by *Proteus mirabilis* (13%) from urinary sources of infection (59%). There was no significant difference in median cefazolin DOTs before (0 days; interquartile range [IQR] 0,0) and after (0 days; IQR 0,0) the breakpoint update (p=0.35). There was no difference in ESBL-producing pathogens (2 vs. 3, p=0.67), *C. difficile* infection (1 vs. 2, p=0.61), in-hospital (3 vs. 7, p=0.20), or 28-day mortality (0 vs. 0, p=1) between the pre- and post-breakpoint update groups.

**Conclusion:**

Updating the breakpoint to a lower value for EKP isolates in the blood did not have an effect on cefazolin use.

**Disclosures:**

**All Authors**: No reported disclosures

